# Moderate NEFA reprogram early follicular development and oocyte competence: evidence for a targetable redox mechanism

**DOI:** 10.3389/fnut.2026.1840637

**Published:** 2026-06-17

**Authors:** Camila Rojo-Fleming, Chiara Di Berardino, Alessia Peserico, Angelo Canciello, Chiara Camerano Spelta Rapini, Gianna Sacchetti, Giulia Capacchietti, Veronica D’Antonio, Donato Angelino, Mauro Serafini, Sebastián Cánovas, Barbara Barboni

**Affiliations:** 1 Department of Bioscience and Technology for Food, Agriculture and Environment, University of Teramo, Teramo, Italy; 2 Department of Physiology, International Excellence Campus for Higher Education and Research (Campus Mare Nostrum), University of Murcia, Murcia, Spain; 3 Biomedical Research Institute of Murcia Pascual Parrilla-IMIB, Murcia, Spain

**Keywords:** antioxidant rescue, early folliculogenesis, lipotoxicity, mitochondrial dysfunction, non-esterified fatty acids (NEFA), oocyte developmental competence, oxidative stress, trolox

## Abstract

**Introduction:**

Elevated circulating non-esterified fatty acids (NEFA), a hallmark of metabolic stress and negative energy balance, are increasingly associated with reduced female fertility. While most studies focus on late oocyte maturation, metabolic disturbances during follicular growth, particularly during the preantral – early antral transition, a critical window for establishing oocyte developmental competence may already impair oocyte quality. Here, we investigated whether sustained, physiologically buffered NEFA elevation during this stage affects oocyte developmental competence.

**Methods:**

Preantral follicles were cultured for 18 days in a three-dimensional ovine *in vitro* folliculogenesis system under physiological (70 μM) or moderately elevated (140 μM) NEFA conditions, with defined fatty acid composition and albumin buffering. The cytoprotective effects of the antioxidants Trolox and resveratrol were also evaluated.

**Results:**

Chronic exposure to moderately elevated NEFA induced a pro-oxidant follicular microenvironment, characterized by reactive oxygen species (ROS) accumulation and oxidative DNA damage, including increased 8-OHdG and mtDNA D-loop oxidation. This stress impaired cumulus cell function and somatic–oocyte communication, reduced oocyte mtDNA copy number and mitochondrial activity, and compromised meiotic and developmental competence despite preserved follicular growth. Antioxidant treatment with Trolox restored mitochondrial function, normalized cumulus activity, and rescued blastocyst development, partially reversing the NEFA-induced phenotype.

**Discussion:**

Prolonged moderate NEFA elevation during early folliculogenesis impairs oocyte competence despite preserved follicular morphology, identifying chronic lipotoxicity and redox imbalance as early and clinically relevant determinants of impaired fertility.

## 1 Introduction

Lipotoxicity refers to the pathological consequences of excessive lipid accumulation in non-adipose tissues, leading to cellular dysfunction and impaired organ homeostasis (1, 2). This condition is primarily driven by increased adipose tissue lipolysis and impaired fatty acid handling and oxidation, resulting in elevated levels of circulating and subsequetly intracellular non-esterified fatty acids (NEFA) (3). This phenomenon is increasingly recognized as part of a broader metabolic–inflammatory imbalance associated with metabolic diseases. Understanding the origins of this imbalance requires consideration of the lifestyle and physiological conditions that drive NEFA mobilization.

Circulating NEFA levels are not only determined by dietary intake but are primarily regulated by adipose tissue lipolysis (4, 5). NEFA increase under conditions of negative energy balance triggering lipolysis, such as fasting, caloric restriction, stress, or adipose tissue insulin resistance (6–8). Consequently, elevated circulating NEFAs often reflect the mobilization of stored fatty acids from adipocytes rather than dietary lipid intake per se.

However, increases in specific saturated fatty acids, particularly palmitic acid (PA), may also occur through *de novo* lipogenesis when energy intake exceeds metabolic demand, especially in the context of high carbohydrate consumption (9). This represents a mechanistically distinct source of palmitate and highlights the importance of distinguishing between total NEFA availability and specific fatty acid origin, as different metabolic pathways may contribute to elevated palmitate levels beyond simple dietary fat intake (9).

The biological impact of NEFA on cell function is not determined solely by their total concentration, as bioavailability and fatty acid composition critically influence lipotoxic outcomes. A key apsect in understanding lipotoxicity is the transport of NEFAs in the bloodstream. Physiologically, albumin acts as a buffering system that sequesters fatty acids; however, when the molar ratio of NEFA exceeds albumin binding capacity, the unbound free fatty acid fraction increases exponentially, thereby amplifying lipotoxic effects at the cellular level in a non-linear manner (6, 7).

When the capacity for fatty acid storage or mitochondrial β-oxidation is exceeded, NEFA accumulate and are redirected toward the synthesis of bioactive lipid intermediates such as diacylglycerols (DAG) and ceramides, a process widely described in metabolically active tissues including liver and skeletal muscle (10). Studies in skeletal muscle cells exposed to saturated fatty acids show that these metabolites impair insulin signaling through inhibition of Akt pathways (11, 12) and contribute to hepatic insulin resistance (10), highlighting the close relationship between nutrient-derived metabolic signals and cellular stress responses. Ceramides also disrupt organelle function, inducing mitochondrial dysfunction and oxidative stress in cardiomyocytes and mouse embryonic fibroblasts (13, 14). Excess fatty acid flux can overload mitochondrial metabolism, promoting incomplete β-oxidation and oxidative stress (15). In parallel, saturated NEFA can alter endoplasmic reticulum membrane composition and activate the unfolded protein response (UPR), contributing to inflammation and apoptosis (16). Together, these events trigger pro-apoptotic signaling and cellular dysfunction, disrupting of tissue homeostasis (3, 17) and linking metabolic stress to immune regulation (18).

In the female reproductive system, exposure to elevated NEFAs has been shown to increase ROS production in follicular cells and oocytes, thereby inducing oxidative stress and compromising oocyte quality in both human (1) and animal models (7). Importantly, the biological effects of fatty acids are strongly dependent on their chemical class. Indeed, while saturated fatty acids such as palmitate and stearate are particularly lipotoxic, inducing endoplasmic reticulum stress and mitochondrial dysfunction, by contrast monounsaturated fatty acids such as oleate may have a buffering or protective role in some contexts by promoting the esterification of fatty acids into triglycerides and sequestering them in lipid droplets (6–8, 17). This mechanistic distinction between saturated fatty acids (SFAs) and monosaturated fatty acids (MUFAs) is critical when designing experimental systems that aim to replicate physiologically relevant lipid exposure patterns.

Alterations in lipid metabolism and elevated circulating NEFAs have been associated with impaired female fertility through both systemic endocrine mechanisms and local ovarian effects. At the systemic level, metabolic dysregulation may affect the hypothalamic–pituitary–ovarian (HPO) axis, leading to altered gonadotropin secretion and disrupted ovarian cyclicity. However, these systemic effects complicate the interpretation of fertility impairments, as they do not allow discrimination between central endocrine dysregulation and direct ovarian toxicity. Increasing evidence indicates that NEFAs directly accumulate within the ovarian follicular environment, where they exert local effects on granulosa cells and the enclosed oocyte (19). Elevated NEFA concentrations in follicular fluid have been associated with reduced oocyte developmental competence in bovine models and have been implicated in subfertility in humans (20–22). Moreover, *in vitro* maturation (IVM) studies have confirmed that short-term NEFA exposure negatively affects isolated oocytes (23).

Despite growing evidence linking altered lipid metabolism to late folliculogenesis/oogenesis outcomes (24–26), the impact of sustained NEFA exposure during the early stages of folliculogenesis remains insufficiently defined (27, 28). *In vivo* studies are frequently confounded by systemic alterations such as obesity, insulin resistance, and chronic inflammation, which preclude direct attribution of reproductive dysfunction to follicle-level NEFA.

Controlled long-term *in vitro* folliculogenesis systems offer a unique opportunity to isolate direct follicular effects of chronic metabolic stress and to investigate how prolonged NEFA exposure affects granulosa–oocyte communication and the acquisition of oocyte competence.

Based on these gaps, the present study aimed to investigate whether sustained exposure to elevated NEFA concentrations directly impairs folliculogenesis and oocyte developmental competence using a long-term three-dimensional (3D) *in vitro* folliculogenesis model in sheep. Preantral follicles (PAfs) isolated from ovine ovaries were cultured ex vivo for 18 days within a biomimetic artificial ovary system based on an electrospun polycaprolactone (PCL) scaffold designed to recapitulate the architecture of the ovarian cortex (19). Two antioxidants with distinct biological properties were evaluated. Trolox, a water-soluble analog of vitamin E, was selected due to its well-established ROS-scavenging activity and compatibility with aqueous culture systems, making it particularly suitable for targeting oxidative stress mechanisms *in vitro* (29, 30). In parallel, resveratrol (RSV), a polyphenolic compound with reported antioxidant and metabolic regulatory properties, was included as a candidate molecule based on previous evidence of its protective effects in reproductive and metabolic contexts (31, 32). Both molecules represent bioactive compounds of nutritional interest capable of modulating oxidative stress pathways. By comparing these antioxidants within a long-term follicle culture model exposed to defined NEFA conditions, this study aimed to determine whether NEFA-induced follicular dysfunction can be prevented or attenuated through targeted modulation of oxidative stress pathways potentially relevant to nutrition-based strategies for maintaining cellular homeostasis and reproductive health.

## 2 Materials and methods

### 2.1 Ethical issues

No ethical concerns were raised, as all biological materials were derived from animal tissues discarded from the food chain in accordance with European Community Regulation (EC) No. 1069/2009. All procedures were performed in an institution authorized by the Italian Ministry of Health (approval number: ABP4169).

### 2.2 Biological sample recovery

#### 2.2.1 Ovary collection

PAfs were isolated from the ovaries of Appenninica breed lambs (33). The ovaries collected at the slaughterhouse were transported to the laboratory in 1 h into a thermostatic container. Upon receipt, they were thoroughly rinsed in a 0.9% NaCl solution supplemented with 1 mg/mL benzoxonium chloride (Cat. No. 032186013 Bialcol Med, Vemedia Pharma S.r.l., Parma, Italy) before further processing. Slices of cortex were obtained before carefully dissecting them into cortical strips of 0.5 × 0.5 × 0.5 cm in HEPES-buffered TCM199 medium (Cat. No. M7528, Sigma-Aldrich).

#### 2.2.2 Isolation and morphological evaluation of PAfs

PAfs were mechanically retrieved from cortical strips using 32G sterile needles under a stereomicroscope within a flowhood. Follicles were classified based on morphological characteristics and size (34). The medium-large PAfs with an average diameter of 245 ± 5 μm (NIS-Elements software of Eclipse Ti Series, Nikon, Tokyo, Japan), intact basal membrane, and compact theca and granulosa layers were then selected for *in vitro* culture (34–36).

### 2.3 PAfs *in vitro* folliculogenesis

The study employed a previously validated three-dimensional culture system (37) designed to support the simultaneous development of multiple PAfs, referred to as the artificial ovary (AO) system. Briefly, the system allows the culture of 10 PAfs per well on an electrospun fibrous scaffold in 233 μL of culture medium.

The scaffold was fabricated from polycaprolactone (PCL) using electrospinning technology to obtain a patterned topology, as previously described (38). The resulting microfibrous structure is characterized by a defined macroporosity (average pore size ~300 μm) and an average fiber diameter of approximately 1 μm. PAfs were cultured using a transwell-based setup, in which the PCL-patterned scaffold was fitted into a U-shaped 48-well plate, enabling a multiple-follicle culture approach (10 PAfs per well) (37).

Long-term *in vitro* PAf culture was performed for 18 days at 38.5 °C in a humidified atmosphere with 5% CO₂. The culture medium consisted of Minimum Essential Medium *α* (αMEM; Cat. No. BE02-002F, Lonza, Basel, Switzerland) supplemented with 5% KnockOut™ Serum Replacement (Cat. No. 10828028, Gibco, Thermo Fisher Scientific), 1% ITS (insulin, transferrin, and selenium; Cat. No. I1884, Sigma), 50 μg/mL ascorbic acid (Cat. No. A4544, Sigma), 2 mM glutamine (Cat. No. BE17-605E/U1, Lonza), and antibiotics (75 mg/L penicillin G and 50 mg/L streptomycin sulfate, Cat. No. DE17-602E, Lonza). The medium was replaced every 48 h and supplemented with 4 IU/mL equine chorionic gonadotropin (eCG; equivalent to 1 μg/mL FOLLIGON^®^, MSD Animal Health S.r.l., Segrate, Italy) (37).

### 2.4 Non-esterified fatty acids supplementation

To generate NEFA: albumin complexes for *in vitro* culture, albumin was used as vehicle a physiological concentration (30 mg/mL) (39, 40). A 3:1 NEFA: albumin molar ratio was considered to mimic the normolipidemic condition (41, 42), while it was increased (> 3:1) to mimic the hyperlipidemic one (41, 42, 118, 120). In this study, bovine serum albumin (BSA; A7030, Sigma-Aldrich) was used. The NEFA pool consisted of oleic acid (OA; HY-N1446), (MedChemExpress), palmitic acid (PA; HY-N0830, MedChemExpress), and stearic acid (SA; HY-B2219, MedChemExpress), selected based on previous studies reporting both the relative composition and physiological concentrations of the predominant circulating NEFAs in ovine serum and follicular fluid (43, 44).

More in detail, OA, PA, and SA were dissolved in ethanol (100 mM, #64–17-5, Merck) and then diluted and incubated at 37 °C for 30 min in αMEM (Cat. No. BE02-002F, Lonza) containing 30 mg/mL BSA to generate NEFA–BSA stock solutions at 1.35, 2.7, and 4.05 mM total NEFA (nNEFA, hNEFA-140, and hNEFA-210), using OA/PA/SA concentrations of 0.54/0.351/0.189, 1.08/0.702/0.378, and 1.62/1.53/0.567 mM, respectively.

Then, NEFA–BSA stock solutions were added to the experimental medium to achieve the desired final concentrations: nNEFA (70 μM total NEFA: 40 μM OA, 20 μM PA, 10 μM SA), hNEFA-140 (140 μM: μM OA, 40 μM PA, 20 μM SA), and hNEFA-210 (210 μM, 120 μM OA, 60 μM PA, 30 μM SA). Of note, the hNEFA-140 and hNEFA-210 concentrations were selected to establish a dose–response curve spanning the range of NEFA levels reported *in vivo* in metabolically stressed ewes (116, 121). The maximum BSA concentration of 1.6 mg/mL was preliminary assessed on long-term *in vitro* culture (Supplementary Figure 1).

### 2.5 Antioxidant supplementation in *in vitro* follicular cultures to counteract NEFA—induced effects

To investigate the influence of antioxidants in mitigating hNEFA effects during the *in vitro* follicular development, two compounds were tested: Resveratrol (RSV, MedChemExpress, HY-16561) and the water-soluble vitamin E analog, Trolox (MedChemExpress, HY-101445). For both antioxidants, dose–response curves were preliminary texted on 3D PAfs covering the range of concentrations previously validated in the literature (29, 45) to evaluate their effects in our experimental model. To this aim, RSV was used at 0, 12.5, 25, 50, and 100 μM (45), while Trolox at 0, 37.5, 75, 150, and 300 μM (29, 46, 111, 117, 119, 122). As reported in Supplementary Figure 2, the highest antioxidant concentrations of 25 and 150 μM for RSV and Trolox, respectively, compatible with a follicular survival above 80% were selected for the experiments.

### 2.6 *In vitro* PAfs culture outcomes

#### 2.6.1 Morphological and functional assessment of *in vitro* follicular growth and development

The *iv*F outcomes were assessed after 18 days by recording follicular growth, and antrum formation and excluding follicles exhibiting degenerative characteristics (loss of 3D microarchitecture, oocyte extrusion, or a darkened somatic compartment). Follicular growth was evaluated by expressing the percentage of diameter increase (∆ growth %). Early antral follicles (EAfs) were categorized as based on the emergence of an initial follicular cavity, on the presence of a translucent oocyte, and well-organized follicular compartments (cumulus and mural layers).

#### 2.6.2 *In vitro* maturation of follicle-enclosed oocytes from EA follicles and oocyte nuclear stage assessment

*In vitro* maturation (IVM) was performed according to previously validated Follicle Enclosed Oocyte (FEO-IVM) system. In detail, the EAfs were cultured on PCL-patterned electrospun scaffolds (47) in 233 μL of in maturation medium, consisting of αMEM supplemented with 20% FBS, 1% glutamine (Cat. No. BE17-605E/U1, Lonza), antibiotics (75 mg/L penicillin-G and 50 mg/L streptomycin sulfate, Cat. No. DE17-602E, Lonza) and 25 IU/mL of human Chorionic Gonadotropin (hCG; equivalent to 6 μg/mL Chorulon^®^, MSD Animal Health S.r.l., Segrate, Italy). The FEO-IVM was carried out at 38.5 °C with 5% CO₂, for 24 h. At the end of FEO-IVM, EAfs were opened with the aid of a stereomicroscope to isolate cumulus oocyte complexes (COCs). The healthy COCs were, then denuded of surrounding cumulus cells and fixed in 4% paraformaldehyde (PFA) for 30 min at room temperature. After fixation, samples were washed in phosphate-buffered saline (PBS) and permeabilized with 0.1% Triton X-100 in PBS for 15 min. Oocytes were then incubated with Hoechst 33342 (#62249, Thermo Fisher Scientific; 5 μg/mL in PBS) for 10 in the dark. Following staining, oocytes were washed in PBS, mounted on glass slides using antifade mounting medium, and examined under a fluorescence microscope equipped with a UV filter set (AxioVision, Carl Zeiss, Jena, Germany). The oocytes were classified according to the meiotic nuclear stages in Germinal Vesicle (GV), Germinal Vesicle Break Down (GVBD/MI), and Metaphase II (MII) oocytes (48).

#### 2.6.3 Oocyte activation

Parthenogenetic activation was carried out accordingly to a previously validated protocol (37) to assess oocyte quality. Briefly, MII oocytes, identified for the extrusion of the first polar body, were activated with ethanol. After 72 h, activated oocytes were fixed and permeabilized in an acetic acid:ethanol solution (1:3 v/v) for at least 12 h. Developmental stages were finally assessed by using 1% Lacmoid staining (Cat. No. 274720, Sigma) under a Phase Contrast Microscope (AxioVert, Carl Zeiss, Jena, Germany).

The developmental competence of MII oocytes was evaluated through *in vitro* fertilization (IVF). IVF was carried out using frozen–thawed semen from rams of proven fertility, following a previously described protocol (49). Briefly, IVF was performed in 50 μL drops at a sperm concentration of 1 × 10^6^ sperm/mL, incubated at 38.5 °C in a humidified atmosphere of 5% CO₂ in air for 20–22 h. Embryo development was assessed at 2 and 8 days of culture. Cleavage and further embryo progression were monitored under an inverted microscope equipped with time-lapse imaging software (NIS-Elements Advanced Research, Version 4.51.00, Eclipse Ti Series, Nikon, Japan), and developmental stages were determined by counting the number of blastomeres. The fertilization rate was calculated as the proportion of cleaved embryos observed on day 2, while the blastocyst rate was determined on day 8 relative to the number of cleaved embryos recorded on day 2.

### 2.7 Molecular analyses

#### 2.7.1 2′,7′-dichlorodihydrofluorescein diacetate assay for reactive oxygen species detection

ROS levels were assessed in exhausted conditioned media collected every 2 days until Day 18, using a spectrofluorometric method based on the 2′,7′-dichlorodihydrofluorescein diacetate (DCFH-DA; ab113851 Abcam) assay. For each time point, a 50 μL aliquot of conditioned medium was incubated with 5 μL of 1 mM DCFH-DA. ROS-induced oxidation of DCFH-DA to the fluorescent compound dichlorofluorescein (DCF) was measured, and fluorescence intensity was recorded at 520 nm (excitation at 480 nm), 2 h after DCFH-DA addition.

#### 2.7.2 8-Hydroxy-2’-deoxyguanosine quantification to assess DNA damage

Oxidized purine nucleotides (e.g., 8-OHdG) are useful markers for the study of DNA damage arising from oxidative stress conditions. DNA oxidative damage was quantified by measuring 8-OHdG using a competitive ELISA kit (ab201734, Abcam), according to the manufacturer’s instructions. Conditioned culture media were collected every 2 days throughout the *in vitro* culture period up to Day 18.

In parallel, follicular lysates were prepared at the end of culture. Follicular walls were mechanically isolated, and DNA was recovered by rinsing the tissue in DNase-free H₂O containing 50 mM Tris–HCl (pH 8.5), 0.5% Tween-20, and 100 μg/mL Proteinase K, as previously described (50–52) Samples were incubated at 55 °C for 16 h to allow enzymatic digestion, followed by heat inactivation of Proteinase K at 95 °C for 10 min. The resulting digests were clarified and used for downstream analyses.

For both conditioned media and follicular lysates, 50 μL aliquots were processed according to the kit protocol. Briefly, samples were incubated with the anti-8-OHdG antibody, transferred to ELISA plates pre-coated with 8-OHdG conjugate, and subjected to competitive binding. After washing, an HRP-conjugated secondary antibody and substrate solution were added. Absorbance was measured at 450 nm using a microplate reader, and 8-OHdG concentrations were calculated from the standard curve and expressed as ng/mL.

#### 2.7.3 Quantitative determination of oxidized DLOOP region produced as damage-associated molecular patterns

Oxidative damage to mitochondrial DNA (mtDNA) is reflected by increased levels of oxidized purine nucleotides, including 8-OHdG. The presence of oxidized purines was evaluated in all experimental conditions following digestion with the formamidopyrimidine-DNA glycosylase (FPG) enzyme and quantified by real-time qPCR targeting the mtDNA D-loop region. By excising oxidized purines, FPG generates abasic sites that impair PCR amplification efficiency, resulting in increased Ct values.

Briefly, DNA was extracted from follicular walls and culture media using the Quick-DNA Microprep Kit (Zymo Research, CA, United States) following manufacturer’s instructions. A 100 ng of genomic DNA (2 μL of stock solution) was incubated for 1 h at 37 °C in a 10 μL reaction mixture containing 1 U of FPG enzyme (New England BioLabs, United Kingdom) together with 10 mM Bis-Tris Propane-HCl, 10 mM MgCl₂, 1 mM DTT, and 0.1 mg/mL BSA. Parallel reactions were also prepared without FPG digestion, serving as negative controls and reference samples for total (non-digested) DNA quantification. Real-time qPCR was then performed to quantify mtDNA using primers targeting the mitochondrially encoded D-loop region. All primers were purchased from Sigma-Aldrich, and their exact sequences are listed in Table 1. The residual non-oxidized D-loop mtDNA levels were expressed as 2^-ΔCt (ΔCT = CT FPG treated − CT FPG untreated) following previously validated workflows (53–55). The percentage of damaged D-loop region was calculated as: *Damage (%) = (1 − amplifiable fraction) × 100.*

**Table 1:** Sequences of primers used in real-time qPCR.

Gene or genomic loci	Forward sequence	Reverse sequence
*SOD2*	5′- TAAACCGTCAGCCTTACAC-3′	5′- ACATTTTCAAACAGTTGCCTA-3′
*SOD3*	5′- CTGGAAGTACCGCTCCAACC-3′	5′- CGCGTTACCGTTCTCCAGAC-3′
*EPHX1*	5′- TTCTACACCCTGACCCTTCC-3′	5′- AGTCATTCAGAGCACAGCC-3′
*GSTA4*	5′-GAAACCCAAGCTCCACTATC-3′	5′-ATCCTGCAACTTCTGCAAC-3′
*GAPDH*	5′-TCGGAGTGAACGGATTTGGC-3′	5′-CCGTTCTCTGCCTTGACTGT-3′
*YWHAZ*	5′-AGACGGAAGGTGCTGAGAAA-3′	5′-CGTTGGGGATCAAGAACTTT-3′
*D-LOOP*	5’-CGCTGAGCCAGTCAGTGT-3’	5’-GGGGAAAGAGTGGGCGATTT-3’
*mtDNA-1* *(10103- 10273 nt)*	5’-CTAGGCCTGTCCCTACTGGT 3’	5’-GCAAGCTGTGAAGTGTGGTG 3’

#### 2.7.4 mtDNA copy number

mtDNA copy number was assessed following DNA isolation from individual oocytes. DNA extraction from single oocytes was carried out according to established protocols (50–52). Briefly, oocytes were incubated in 5 μL of lysis buffer containing 50 mM Tris–HCl (pH 8.5), 0.5% Tween-20, and 100 μg/mL Proteinase K at 55 °C for 16 h, followed by enzyme inactivation at 95 °C for 10 min. Ovine-specific primers targeting the mtDNA region between nucleotides 10,103 and 10,273 were used, and absolute mtDNA copy number was determined by standard curve-based quantification (56).

#### 2.7.5 Assessment of mitochondrial membrane potential (JC-1 staining)

Mitochondrial membrane potential (ΔΨm) was evaluated using the JC-1 probe (#65–0851, Thermo Fisher Scientific) according to Cotterill et al. (57), with minor modifications. Briefly, following follicle retrieval and mechanical isolation of cumulus–oocyte complexes (COCs), oocytes were denuded and incubated with JC-1 (1 μg/mL) for 30 min at 37 °C in the dark. After 15 min of incubation, DAPI (D9542, Sigma-Aldrich) was added to counterstain nuclei. Oocytes were then washed twice in PBS containing 0.05% BSA and immediately analyzed without fixation using a Nikon confocal microscope (Nikon AR1, Düsseldorf, Germany) equipped with NIS-Elements software version 4.40 (Nikon, Düsseldorf, Germany). JC-1 was excited at 488 nm. Emission of JC-1 monomers, indicative of low mitochondrial polarization (low ΔΨm), was detected using a green filter (520–527 nm), whereas emission of JC-1 aggregates, reflecting high mitochondrial polarization (high ΔΨm), was detected using an orange filter (590 nm).

#### 2.7.6 RNA extraction and cDNA synthesis for gene expression assay

The total RNA was extracted from follicular walls with a Total RNA Purification Kit (Norgen Biotek Corp. SKU-17200) following the manufacturer’s instructions. A total of 1 μg of total RNA was retrotranscribed using oligodT primers (Bioline, BIO-38029) and Tetro Reverse Transcriptase (Bioline, BIO-65050), following the manufacturer’s instructions.

#### 2.7.7 Real-time qPCR

The qPCRs were carried out using the SensiFAST SYBR Lo-ROX kit (Bioline BIO-94050) on a QuantStudio 3 Real-Time PCR System (Life Technologies, Carlsbad, CA, United States), according to the manufacturer’s instructions. The following PCR conditions were used for all the experiments: 95 °C for 2 min for cDNA and 3 min for DNA, followed by 40 cycles at 95 °C for 10 s and 60 °C for 30 s. For gene expression analysis, relative quantification was performed by using the ∆Ct method. GAPDH (Glyceraldehyde 3-phosphate dehydrogenase) and YWHAZ (Tyrosine 3-Monooxygenase/Tryptophan 5-Monooxygenase Activation Protein Zeta) were selected among the housekeeping genes for gene quantification. The expression profiles were similar with both reference genes. The primer sequences are reported in Table 1.

#### 2.7.8 Immunofluorescence

After denudation, cumulus cells from the corresponding NEFA culture groups were collected and washed in PBS. Cells were fixed in 4% PFA for 30 min at room temperature, washed three times in PBS, and permeabilized with 0.1% Triton X-100 in PBS for 10 min. Following three washes in PBS, samples were blocked in PBS containing 1% BSA and 0.1% Tween-20 for 1 h at room temperature. Cells were incubated overnight at 4 °C in the dark with mouse monoclonal anti-HAS2 antibody (#AB140671, Abcam) diluted in PBS with 1% BSA and 0.01% Tween-20. After washing (3 × 5 min in PBS/0.01% Tween-20), samples were incubated for 1 h at room temperature with CY3-conjugated anti-mouse secondary antibody (#A-11032, Alexa Fluor 594) in PBS/1% BSA. Nuclei were counterstained with DAPI (D9542, Sigma-Aldrich) for 10 min, followed by three washes in PBS and a final rinse in distilled water. Samples were mounted and imaged with a Nikon confocal microscope (Nikon Arl, Düsseldorf, Germany) equipped with NIS-Element software 4.40 (Nikon, Düsseldorf, Germany). In all experiments, non-immune serum was used in place of the primary antisera as a negative control (58). All controls performed were negative.

### 2.8 Statistical analysis

Three independent experimental replicates were performed to evaluate intra-experimental variability. The exact values of n (representing the sample size) and the statistical significance are provided in the captions of the figures. The data are presented as the percentage or mean ± SD. Statistical analyses were performed using GraphPad Prism 9 (GraphPad Software). Two-way ANOVA was used to evaluate the effects of multiple factors, followed by appropriate *post-hoc* tests (Šídák’s, Dunnett’s, Tukey’s, or Fisher’s LSD) depending on the experimental design and comparisons of interest. Statistical significance was set at *p* < 0.05.

## 3 Results

### 3.1 Dose-dependent effects of NEFA uncouple follicular growth from oocyte meiotic and developmental competence

To determine whether increasing NEFA concentrations affect follicular development and oocyte competence, follicles were cultured without NEFA (CTR) under physiological NEFA conditions (nNEFA) or exposed to 2x (hNEFA-140) or 3x (hNEFA-210 μM) NEFA concentrations. Exposure to the highest NEFA concentration (hNEFA-210) was early incompatible with follicle viability. Clear signs of follicular degeneration were observed in the majority of follicles after 8 days of ivF culture, leading to premature termination of this condition (Supplementary Figure 3). Accordingly, hNEFA-210 follicles were excluded from subsequent long-term analyses.

**Figure 1:**
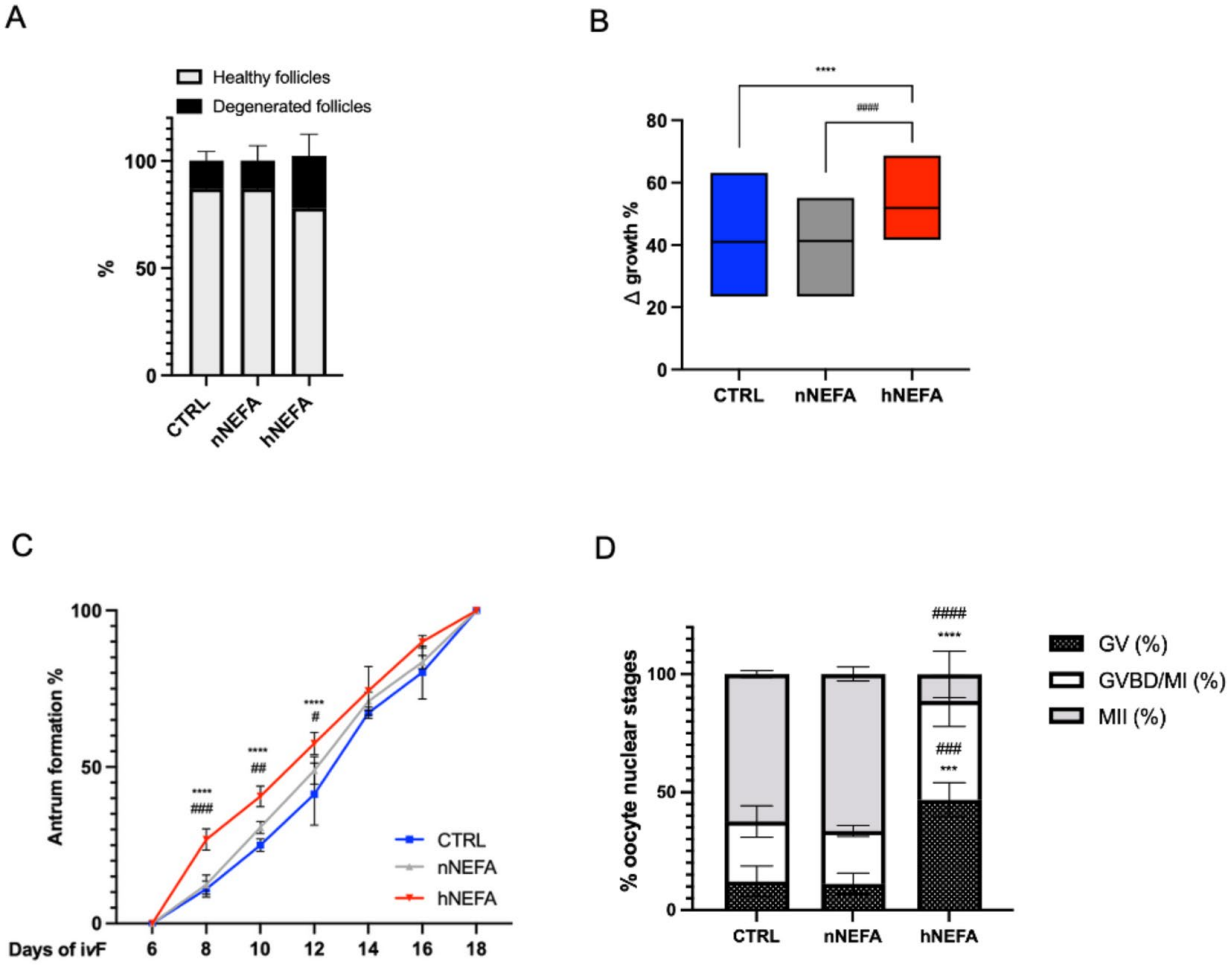
NEFA exposure and its impact on *in vitro* follicular development and oocyte maturation. Exposure to high NEFA concentrations differentially affects follicular growth and oocyte competence during long-term *in vitro* follicle culture. **(A)** Morphological evaluation of follicular health and degeneration at the end of *18 days of IV*F culture. hNEFA-210 cultures were terminated at day 8 due to complete follicle degeneration (see Supplementary Figure 2); therefore, no day-18 data are available for this group. **(B)** Representative graph showing follicular *Δ* growth rate after 18 days of *in vitro* culture. **(C)** Time-course of antrum formation throughout the 18-day culture period. **(D)** Percentage distribution of oocytes across nuclear maturation stages (GV, GVBD/MI, MII). Data (mean ± SD) represent a total of 240 follicles pooled from three independent biological replicates. Statistical significance was assessed using two-way ANOVA followed by Tukey’s *post-hoc* test (****p < 0.0001). Where not indicated by curly brackets, multiple comparisons are defined as follows (^*^p < 0.05, ^**^p < 0.01, ^***^p < 0.001, ^****^p < 0.0001 for CTRL vs. hNEFA; ^#^*p* < 0.05, ^##^*p* < 0.01, ^###^*p* < 0.001, ^####^*p* < 0.0001 for nNEFA vs. hNEFA). Only significant differences are indicated.

At lower NEFA concentrations, follicle survival was largely preserved. In particular, hNEFA-140 supported follicular development with only a slightly increased final degeneration rate compared to CTRL and nNEFA conditions (Figure 1A). Follicles cultured under CTRL, nNEFA, and hNEFA-140 conditions exhibited comparable survival throughout the culture period.

Despite preserved viability, hNEFA-140 significantly affected follicular growth dynamics. These follicles (hNEFA-140) displayed a greater increase in diameter compared to CTRL and nNEFA conditions (*Δ* growth from day 0: CTRL 41.0%, nNEFA 40.8% vs. hNEFA-140 52.3%; *p* < 0.0001; Figure 1B). Consistently, hNEFA-140 also accelerated antrum formation during the culture period (Figure 1C). Oocytes reached a fully grown diameter in all experimental groups, with no significant differences in final oocyte size (CTRL: 114.2 ± 4.5 μm; nNEFA: 117.0 ± 4.5 μm; hNEFA-140: 117.3 ± 4.3 μm: *p* > 0.05).

In contrast to follicular growth, the acquisition of oocyte competence was markedly impaired under hNEFA-140 conditions. A significantly higher proportion of oocytes failed to resume meiosis, remaining at the germinal vesicle (GV) stage (46.8% in hNEFA-140 *vs*. 11.2% in CTRL and 12.2% in nNEFA; *p* < 0.001). Accordingly, the percentage of metaphase II (MII) oocytes was strongly reduced in the hNEFA-140 group too (11.3% *vs.* 62.6 and 66.5%, in CTRL and nNEFA, respectively; *p* < 0.0001; Figure 1D).

Parthenogenetic activation further confirmed the compromised developmental competence of hNEFA-140–derived oocytes. MII oocytes obtained under hNEFA-140 conditions completely failed to undergo activation (100% non-cleaved embryos; *p* < 0.0001), whereas CTRL and nNEFA oocytes showed comparable activation rates (>80%) and similar cleavage progression, including embryos with more than eight nuclei (39.8 and 43.0%, respectively; Table 2).

**Table 2:** Effects of NEFA exposure on developmental competence.

Experimental Group	MII oocytes (n°)	Parthenogenetic embryo development			
		Uncleaved (%; SD)	PN (%; SD)	<8 nuclei (%; SD)	> 8 nuclei (%; SD)
CTRL	120	11.8 ± 0.9****	24.7 ± 5.7****	23.7 ± 2.8****	39.8 ± 7.3****
nNEFA	120	12.2 + 1^####^	20.3 + 6^####^	24.5 + 3.2^####^	43 + 6.4^####^
hNEFA	26	100 + 0	0 + 0	0 + 0	0 + 0

Cleavage stage distribution after MII oocyte parthenogenetic activation. Three independent biological replicates were performed. Statistical significance was assessed using two-way ANOVA followed by Tukey’s *post-hoc* test (****p < 0.0001). Multiple comparisons are defined as follows (^*^p < 0.05, ^**^p < 0.01, ^***^p < 0.001, ^****^p < 0.0001 for CTRL vs. hNEFA; ^#^*p* < 0.05, ^##^*p* < 0.01, ^###^*p* < 0.001, ^####^*p* < 0.0001 for nNEFA vs. hNEFA). Only significant differences are indicated.

Collectively, these morpho-functional readouts indicate that exposure to hNEFA-140 supports follicular growth while markedly impairing the acquisition of oocyte meiotic and developmental competence. By contrast, CTRL and nNEFA conditions yielded comparable outcomes. Based on these observations, nNEFA was selected as the reference control condition for all subsequent experiments.

### 3.2 Sustained hNEFA exposure establishes an oxidative, DNA-damaging follicular environment and impairs oocyte mitochondrial integrity

To investigate whether prolonged exposure to moderately elevated NEFA levels alters the redox balance of the follicular environment, oxidative stress–related parameters were analyzed with a multifactoral approach under hNEFA-140 conditions (hereafter referred to as hNEFA).

**Figure 2:**
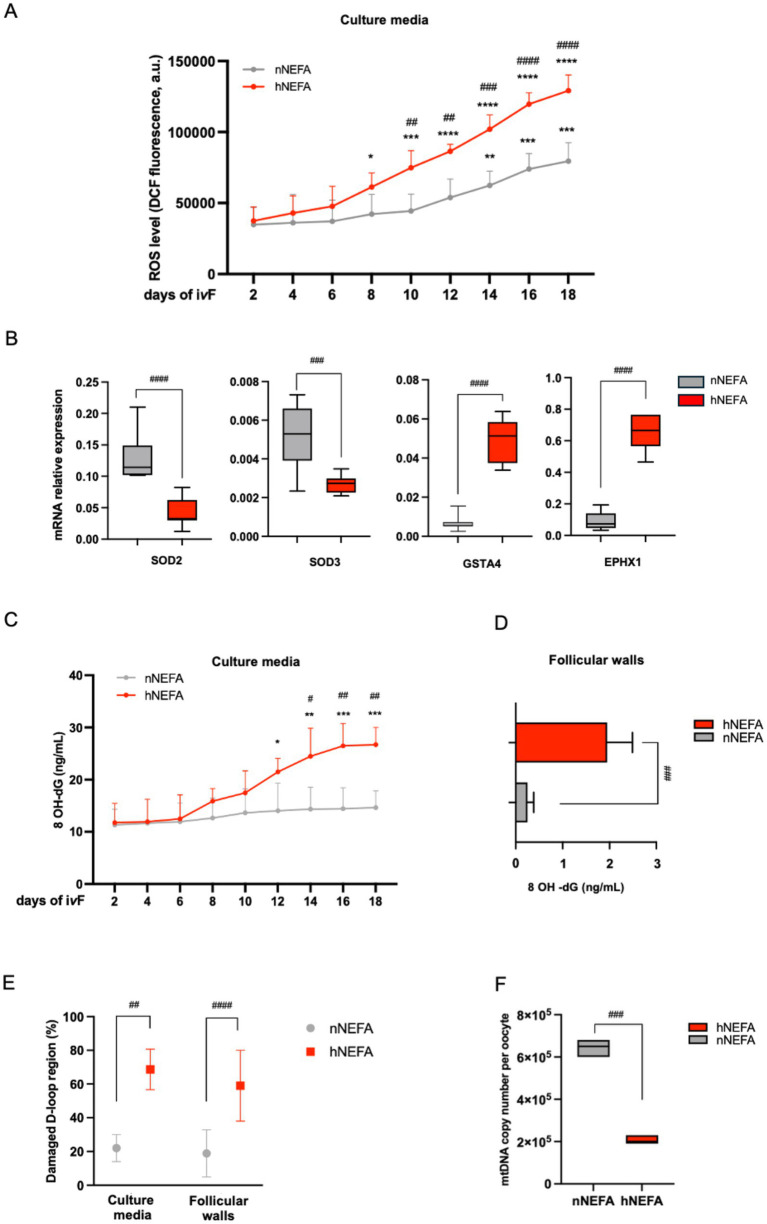
Sustained hNEFA exposure induces oxidative stress, nuclear and mitochondrial DNA damage, and reduces oocyte mitochondrial biogenesis. **(A)** Time course of reactive oxygen species (ROS) production measured in culture media by DCF-DA fluorescence during *in vitro* culture**. (B)** Relative mRNA expression levels of antioxidant genes (*SOD2*, *SOD3*, *GSTA4*, and *EPHX1*) in follicular walls from the different experimental groups, quantified using the ΔCt method. **(C)** Quantification of 8-OHdG levels in culture media over an 18 -day *in vitro* culture period using an 8-OHdG ELISA kit, as an indicator of oxidative DNA damage. **(D)** Quantification of 8-OHdG levels in follicular walls at the end of the 18 day *in vitro* culture using an 8-OHdG ELISA kit. **(E)** Oxidation of the mtDNA D-loop region, expressed as percentage. Quantification of undamaged mtDNA D-loop was performed by qPCR and normalized to nuclear DNA using the ΔCt method (ΔCt raw data are included in Supplementary Figure 4). Data ΔCt data were used to calculate the percentage of oxidized mtDNA, as detailed in the Materials and Methods. **(F)** mtDNA copy number in single oocytes quantified by qPCR. Data (mean ± SD) represent a total of 90 follicles pooled from three independent biological replicates. Statistical significance was assessed using two-way ANOVA followed by Fisher’s test (*p < 0.05, **p < 0.01, ***p < 0.001, ****p < 0.0001 vs. day 2) or Šídák’s test (^#^*p* < 0.05, ^#^*p* < 0.01, ^###^*p* < 0.001, ^###^*p* < 0.0001 for nNEFA vs. hNEFA). *t*-test was applied for **(B,D,F)** (^##^*p* < 0.01, ^###^*p* < 0.001, ^###^*p* < 0.0001 for nNEFA vs. hNEFA). Only significant differences are indicated.

A time-dependent increase in extracellular ROS levels was detected in hNEFA culture media, as measured by DCF-DA fluorescence. ROS accumulation became significant from day 8 of *in vitro* follicle culture and progressively increased thereafter, indicating sustained oxidative pressure over time (Figure 2A).

Consistent with this oxidative environment, analysis of antioxidant and detoxification gene expression in follicular walls revealed significant alterations under hNEFA conditions. Mitochondrial (SOD2) and extracellular (SOD3) superoxide dismutases were significantly downregulated compared with nNEFA condition (*p* < 0.05), whereas stress-response and detoxification markers GSTA4 and EPHX1 were significantly upregulated (*p* < 0.05; Figure 2B).

The presence of oxidative damage was further assessed at DNA level. hNEFA exposure significantly increased the levels of 8-hydroxy-2′-deoxyguanosine (8-OHdG), a biomarker of oxidative DNA injury, both in conditioned media (26 vs. 14 ng/mL; *p* < 0.0001; Figure 2C) and in follicular walls at day 18 of culture (1.95 vs. 0.25 ng/mL; *p* < 0.0001; Figure 2D).

Mitochondrial DNA integrity was similarly affected. hNEFA induced a significant increase in the fraction of oxidized mtDNA D-loop regions, detected both in conditioned media (≈3-fold increase; *p* < 0.05) and in follicular walls (≈3.3-fold increase; *p* < 0.01) (Figure 2E and Supplementary Figure 4). Finally, analysis of the germinal compartment revealed that hNEFA exposure significantly reduced mitochondrial DNA copy number in single oocytes. Oocytes derived from hNEFA-cultured follicles displayed an approximately 3-fold reduction in mtDNA content compared with nNEFA controls (2.0 × 10^6^ vs. 6.4 × 10^6^ copies per oocyte; *p* < 0.001; Figure 2F).

### 3.3 Antioxidant targeting of oxidative stress reveals a Trolox-specific rescue of hNEFA-induced follicular and oocyte dysfunction

To assess whether oxidative stress contributes to the functional defects induced by hNEFA exposure, follicles were cultured under hNEFA conditions in the presence of either a pharmacological antioxidant (resveratrol, RSV) or a nutritional one (Trolox).

**Figure 3:**
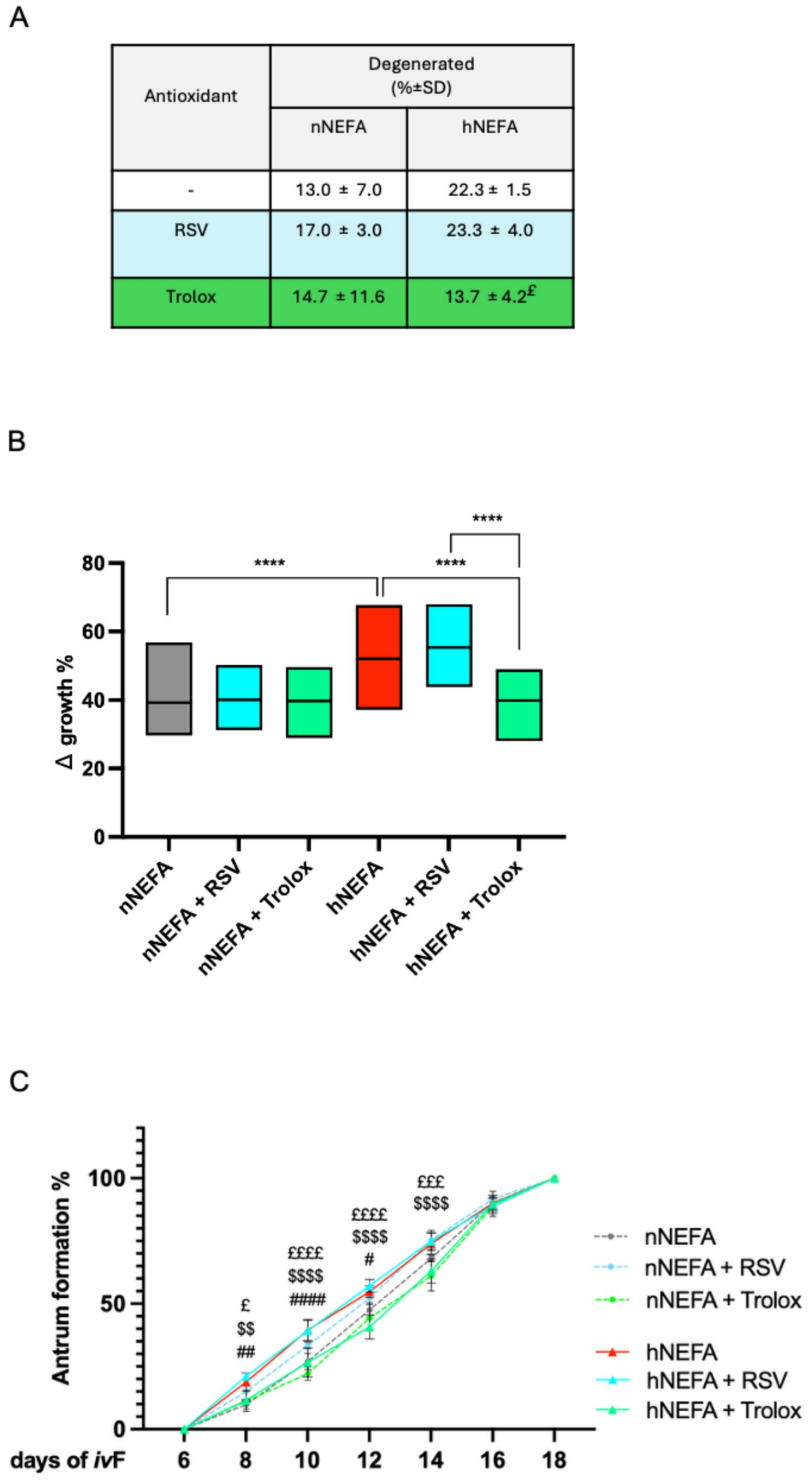
Trolox, but not resveratrol, rescues hNEFA-induced alterations in follicular growth and antrum formation. **(A)** Morphological evaluation of follicular degeneration, **(B)** Follicular Δ growth rate, and **(C)** Antrum formation at the end of an 18 day of culture under nNEFA or hNEFA conditions, in the presence or absence of 25 μM RSV or 150 μM Trolox. Data (mean ± SD) represent a total of 120 follicles pooled from three independent biological replicates. Statistical significance was assessed using one-way ANOVA followed by two-way ANOVA followed by Tukey’s *post-hoc* test (****p < 0.0001). Where not indicated by curly brackets, multiple comparisons are defined as follows (^#^*p* < 0.05, ^##^*p* < 0.01, ^####^*p* < 0.0001 for nNEFA vs. hNEFA; ^$^*p* < 0.05, ^$$$$^*p* < 0.0001 for nNEFA + Trolox vs. hNEFA + RSV; ^£^*p* < 0.05, ^££££^*p* < 0.0001 for hNEFA + Trolox vs. hNEFA). Only statistically significant differences are indicated.

Among the two antioxidants tested, only Trolox significantly improved follicular morpho-functional parameters under hNEFA conditions. Trolox supplementation reduced the incidence of follicular degeneration compared with hNEFA alone (Figure 3A). In addition, it prevented the hNEFA-associated increase in follicular growth rate (%*Δ* growth from day 0: nNEFA 40.0% vs. hNEFA 52.8% vs. hNEFA + Trolox 39.4%; *p* < 0.0001; Figure 3B) and buffered the acceleration of antrum formation observed under hNEFA exposure (*p* < 0.05; Figure 3C). Notably, neither Trolox nor RSV affected follicular survival, growth, or antrum formation when follicles were cultured under nNEFA conditions (Figure 3C), indicating that antioxidant supplementation did not alter follicular dynamics in the absence of metabolic stress.

**Figure 4:**
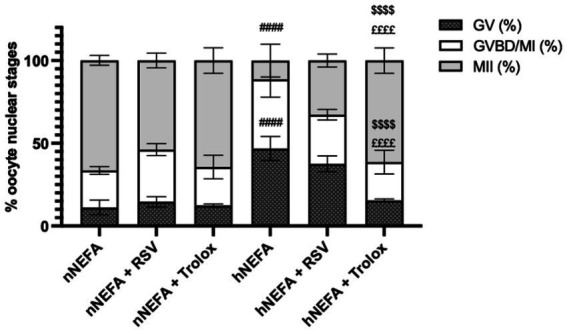
Trolox restores meiotic and developmental competence of oocytes exposed to hNEFA during long-term follicle culture. Distribution of oocytes across nuclear maturation stages (GV, GVBD/MI, and MII) following the FEO–IVM protocol applied to oocytes derived from long-term *in vitro* follicle culture under nNEFA or hNEFA conditions, in the presence or absence of RSV (25 μM) or Trolox (150 μM). Data are presented as mean ± SD and represent a total of 300 follicles pooled from three independent biological replicates. Statistical significance was assessed using two-way ANOVA followed by Tukey’s *post-hoc* test (^#^*p* < 0.05, ^####^*p* < 0.0001 for hNEFA vs. nNEFA; ^£^*p* < 0.05, ^££££^*p* < 0.0001 for hNEFA + Trolox vs. hNEFA; ^$^*p* < 0.05, ^$$$$^*p* < 0.0001 for nNEFA + Trolox vs. hNEFA). Only statistically significant differences are indicated.

The beneficial effect of Trolox under hNEFA conditions extended to oocyte quality. Trolox supplementation markedly increased the proportion of metaphase II (MII) oocytes compared with hNEFA alone (66.4% *vs.* 11.3%; *p* < 0.0001; Figure 4), whereas RSV failed to restore meiotic competence in hNEFA-exposed oocytes (Figure 4).

Consistently, Trolox also restored the developmental competence of hNEFA-derived MII oocytes. Parthenogenetic activation rates reached approximately 82%, and embryo development progressed to advanced cleavage stages (>8 nuclei; 36%) under hNEFA + Trolox conditions, achieving performances comparable to those of nNEFA-derived oocytes (36% vs. 42%; *p* < 0.0001; Table 3A). In contrast, RSV supplementation did not improve developmental outcomes under hNEFA conditions (Table 3A).

**Table 3:** **(A)** Distribution of cleavage stages following parthenogenetic activation of MII oocytes, **(B)** developmental competence of oocytes derived from the three experimental groups after *in vitro* fertilization (IVF).

(A)					
Experimental Group	MII oocytes (n°)	Parthenogenetic embryo development			
		Uncleaved (%; SD)	PN (%; SD)	<8 nuclei (%; SD)	> 8 nuclei (%; SD)
nNEFA	100	12.7 ± 2^#^	21.3 ± 6^#^	23 ± 3.2^#^	42 ± 5.4^#^
nNEFA + RSV	100	11 + 3	26 + 6.5	25 + 3.2	38 + 6.4
nNEFA + Trolox	100	10 + 6$	22 + 4.1^$^	28 + 6.0^$^	40 + 6^$^
hNEFA	26	100 + 0	0 + 0	0 + 0	0 + 0
hNEFA + RSV	100	100 + 0	0 + 0	0 + 0	0 + 0
hNEFA + Trolox	100	18 + 4^£^	20 + 3^£^	26 + 1.6^£^	36 + 2.3^£^

(B)				
Experimental Group	MII oocytes (n°)	Post-fertilization embryo development		
		Activation rate (%; SD)	*n°*	Blastocyst rate (%; SD)
nNEFA	75	52.2 ± 3.6	38	6.5 ± 2.9
hNEFA	30	–	0	–
hNEFA + Trolox	74	49.6 ± 5	34	5.2 ± 3.2

The activation rate was calculated based on the number of cleaved embryos at Day 2 and the embryo development, expressed as blastocyst rate, was calculated by analyzing the reached stage at Day 8 and calculated (based on the number of cleaved embryos recorded at Day 2). Three independent biological replicates were performed. Statistical significance was assessed using two-way ANOVA followed by Tukey’s *post-hoc* test (^#^*p* < 0.05, ^#^###*p* < 0.0001 for hNEFA vs. nNEFA; ^£^*p* < 0.05, ^££££^*p* < 0.0001 for hNEFA + Trolox vs. hNEFA; ^$^*p* < 0.05, ^$$$$^*p* < 0.0001 for nNEFA + Trolox vs. hNEFA). No significant differences were found between nNEFA and hNEFA+trolox (*p* > 0.05).

To further evaluate whether oxidative imbalance induced by NEFA exposure affected oocyte developmental competence, *in vitro* fertilization (IVF) assays were performed, by focusing specifically on the Trolox condition, which had previously demonstrated the highest efficacy in restoring meiotic competence and parthenogenetic development. To this aim, mature oocytes derived from nNEFA, hNEFA, and hNEFA + Trolox cultural conditions were used (Table 3B).

In the nNEFA group, 75 MII oocytes were obtained, with an activation rate of 52.2 ± 3.6%, yielding 38 embryos and a blastocyst rate of approximately 6%. In contrast, exposure to hNEFA markedly impaired developmental competence: only 30 MII oocytes were recovered and no post-fertilization development was observed, with complete absence of activated embryos.

Notably, Trolox supplementation substantially restored fertilization and embryonic development under lipotoxic conditions. The hNEFA + Trolox group yielded 75 MII oocytes, with an activation rate of 49.6%, resulting in 34 embryos and a blastocyst rate of approximately 5%, values comparable to those observed in the nNEFA group (Table 3B).

**Figure 5:**
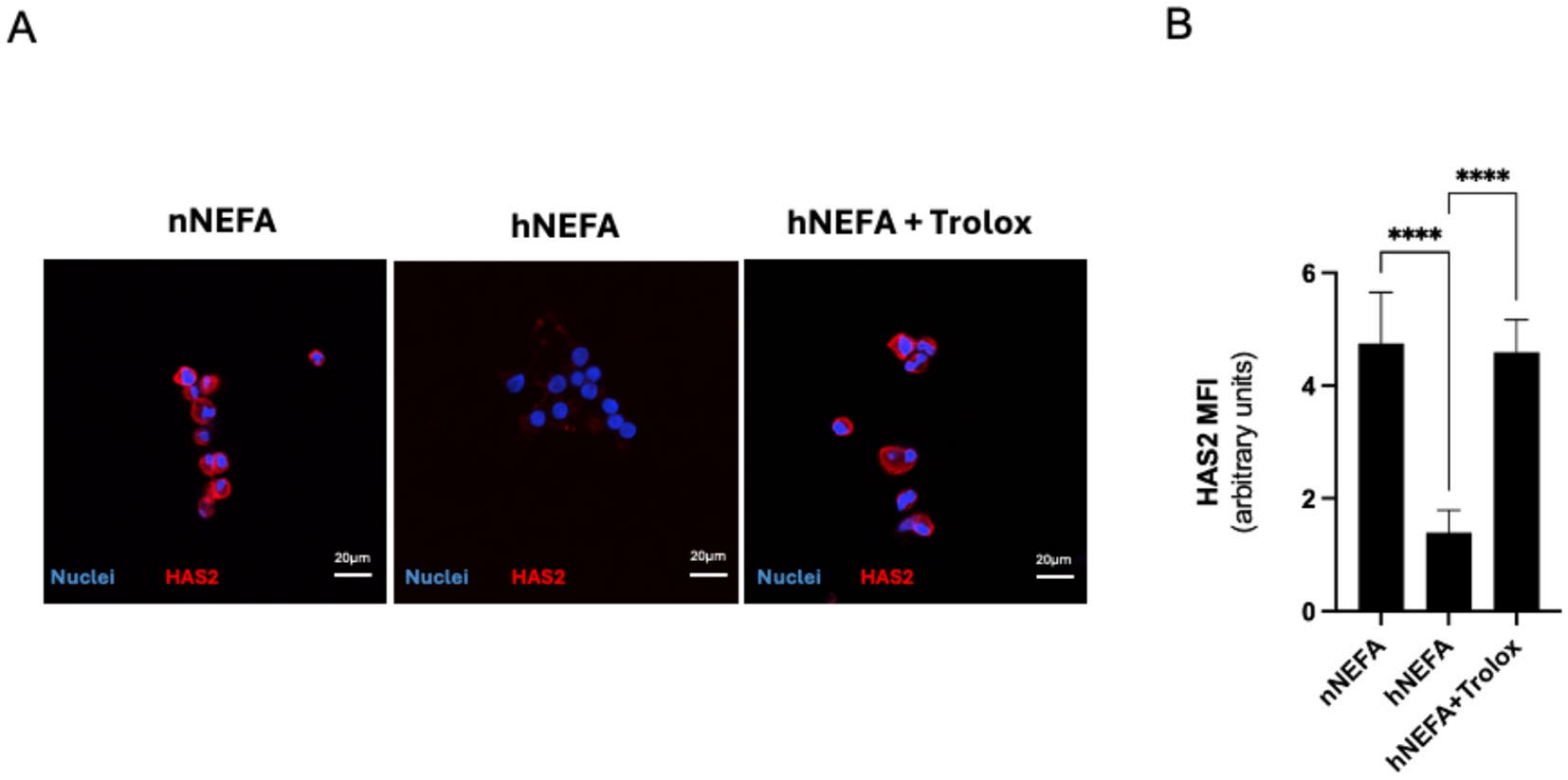
hNEFA condition disrupts cumulus HAS2 expression, which is restored by Trolox. **(A)** Representative confocal images **(B)** and quantification of Alexa-Fluor 594 (HAS2) in cumulus cells. Nuclei are counterstained with Hoechst. At least 30 cells were quantified per condition. Three independent biological replicates were performed. Statistical analysis was performed using one-way ANOVA followed by Tukey’s *post-hoc* test (^****^p < 0.0001).

To explore whether the observed impairment involved alterations in cumulus cell function and somatic–germline communication, HAS2 expression was evaluated as a marker of cumulus expansion and oocyte competence. hNEFA exposure significantly reduced HAS2 expression in cumulus cells compared with nNEFA conditions (*p* < 0.0001). Trolox supplementation preserved HAS2 expression under hNEFA conditions, restoring levels comparable to nNEFA (Figures 5A,B).

Collectively, these data indicate that hNEFA exposure compromises oocyte fertilization capacity and early embryonic development, while Trolox supplementation restores both functional competence and cumulus cell HAS2 expression under lipotoxic conditions.

### 3.4 Trolox attenuates hNEFA-induced oxidative damage, restores follicular mitochondrial integrity and rescues oocyte mitochondrial function despite reduced mtDNA copy number

**Figure 6:**
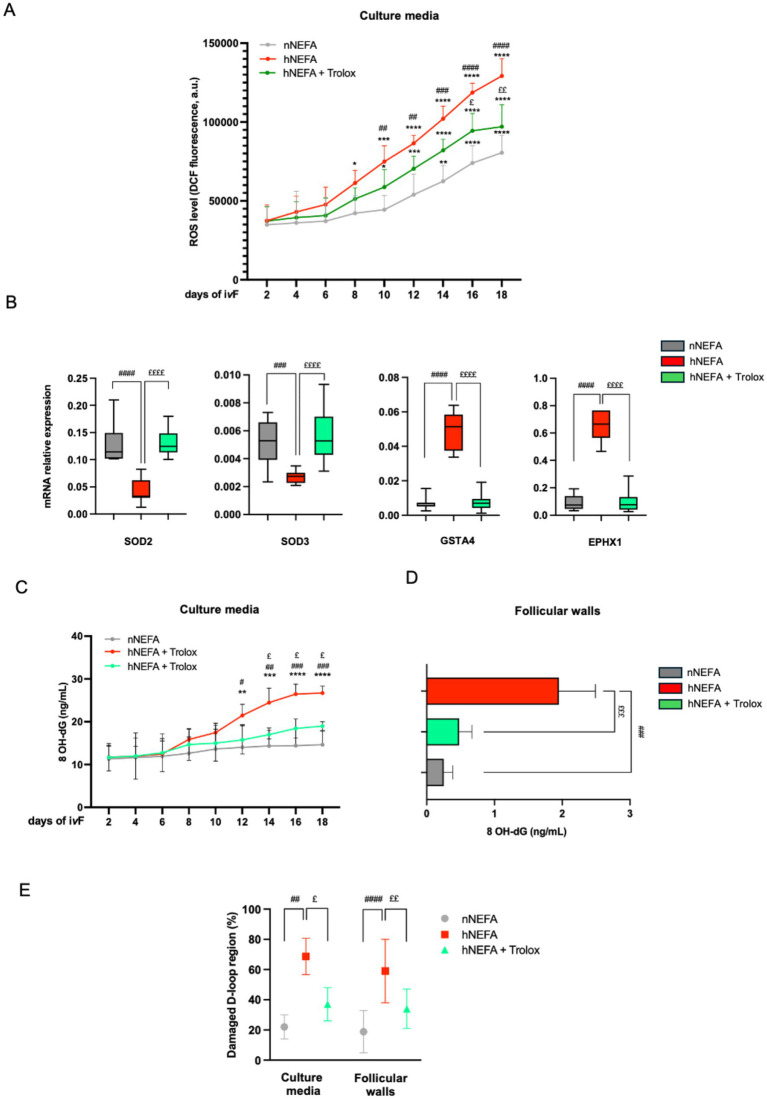
Trolox reduces oxidative stress and restores nuclear and mitochondrial DNA integrity in the somatic compartment under hNEFA conditions. **(A)** Time course of reactive oxygen species (ROS) production measured in culture media by DCF-DA fluorescence during *in vitro* culture. **(B)** Relative mRNA expression levels of antioxidant genes (SOD2, SOD3, GSTA4, and EPHX1) in follicular walls from the different experimental groups, quantified using the ΔCt method. **(C)** Quantification of 8-OHdG levels in culture media over an 18 day *in vitro* culture period using an 8-OHdG ELISA kit, as an indicator of oxidative DNA damage. **(D)** Quantification of 8-OHdG levels in follicular walls at the end of the 18-day *in vitro* culture using an 8-OHdG ELISA kit. **(E)** Oxidation of the mtDNA D-loop region, expressed as percentage. Quantification of undamaged mtDNA D-loop was performed by qPCR and normalized to nuclear DNA using the ΔCt method (ΔCt raw data are included in Supplementary Figure 5). ΔCt data were used to calculate the percentage of oxidized mtDNA, as detailed in the Materials and Methods. Data (mean ± SD) represent a total of 90 follicles pooled from three independent biological replicates. Statistical significance was assessed using two-way ANOVA followed by Dunnett’s test (*p < 0.05, **p < 0.01, ***p < 0.001, ****p < 0.0001 vs. day 2) or Tukey’s test (^#^*p* < 0.05, ^##^*p* < 0.01, ^###^*p* < 0.001, ^####^*p* < 0.0001 for hNEFA vs. nNEFA; ^£^*p* < 0.05, ^££^*p* < 0.01, ^£££^*p* < 0.001, ^££££^p < 0.0001 for hNEFA vs. hNEFA + Trolox). Only significant differences are indicated.

Trolox supplementation induced a time-dependent reduction in extracellular ROS levels in the culture medium of hNEFA. Specifically, compared with untreated hNEFA, ROS accumulation began to decrease from day 8 of culture (Figure 6A).

This reduction became statistically significant during the late culture period, with ROS levels significantly lower from day 16 onward (*p* < 0.05). At this stage, ROS levels in Trolox-treated hNEFA were significantly lower than in untreated hNEFA and reached values comparable to those observed in nNEFA.

Consistent with this reduction in oxidative burden, Trolox markedly modulated the expression of redox-related genes in the somatic follicular compartment. Specifically, both mitochondrial and extracellular superoxide dismutases (SOD2 and SOD3) were significantly upregulated compared with hNEFA conditions (>2-fold increase; *p* < 0.0001), whereas genes encoding detoxification enzymes (GSTA4 and EPHX1) were strongly downregulated (16- and 12-fold reduction *vs.* hNEFA, respectively; *p* < 0.0001), with expression levels approaching those observed under nNEFA conditions (Figure 6B).

Levels of 8-OHdG in culture media were significantly reduced by Trolox supplementation from day 14 onward compared with hNEFA alone (*p* < 0.05), reaching values comparable to those measured under nNEFA conditions at the end of the culture period (19.0 vs. 14.6 ng/mL; *p* > 0.05; Figure 6C).

Similarly, in follicular walls, Trolox markedly reduced 8-OHdG accumulation on DNA compared with hNEFA alone (1.95 vs. 4.7 ng/mL; *p* < 0.001), fully restoring levels to those observed in nNEFA follicles (*p* > 0.05; Figure 6D).

**Figure 7:**
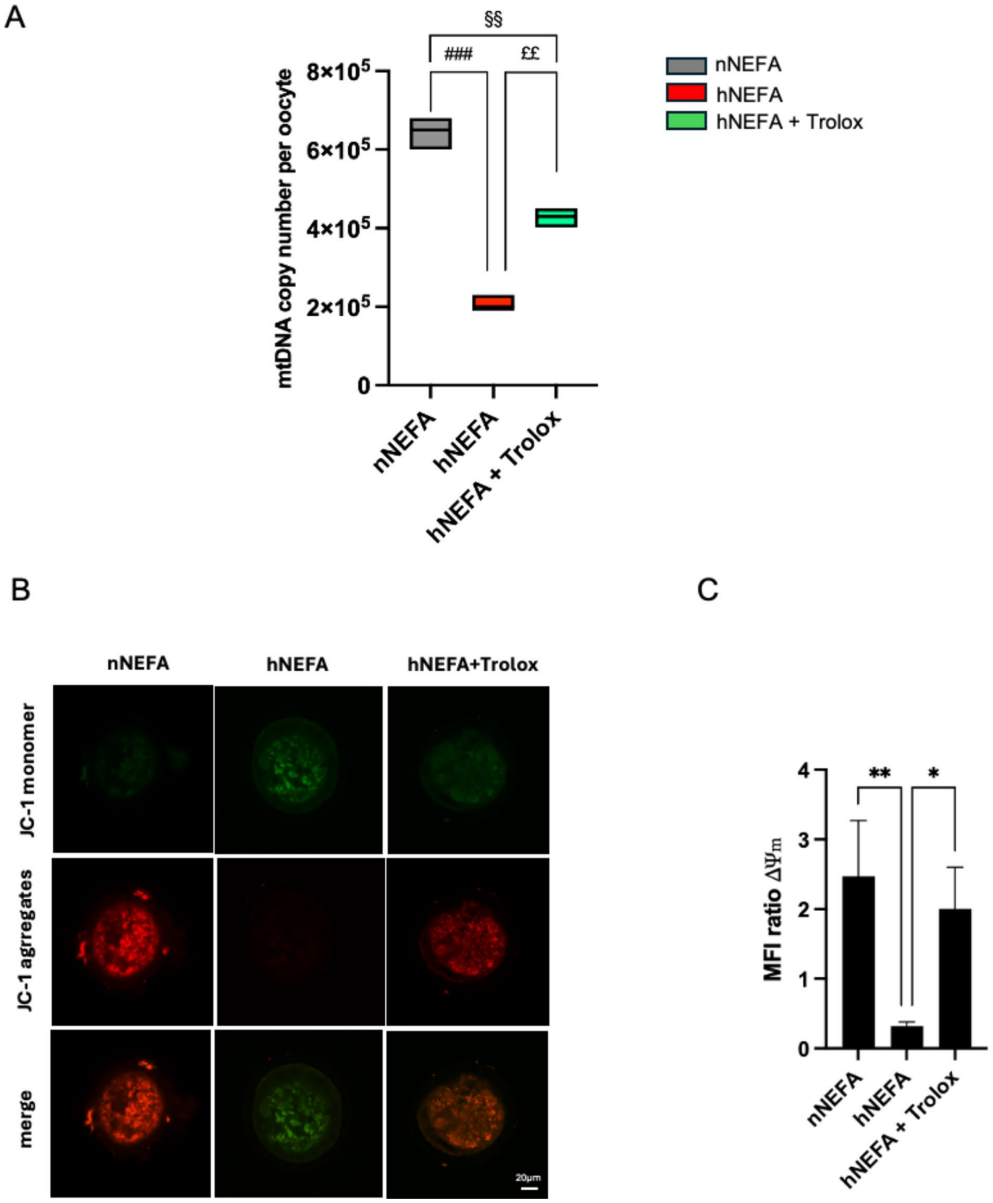
Trolox rescues mitochondrial activity despite incomplete mtDNA restoration in hNEFA exposed oocytes. **(A)** mtDNA copy number in single oocytes quantified by qPCR. Representative images **(B)** and quantification **(C)** of JC-1 staining as a measure of the mitochondrial membrane potential. Mitochondrial membrane potential was evaluated using the JC-1 probe. Red fluorescence indicates polarized mitochondria (JC-1 aggregates), whereas green fluorescence reflects depolarized mitochondria (JC-1 monomers). The mean fluorescence intensity (MFI) was analyzed in oocytes and expressed as MFI ratio between red and green signals. Data (mean ± SD) represents a total of 90 follicles pooled from three independent biological replicates. Statistical significance was assessed using two-way ANOVA followed by Dunnett’s test (*p < 0.05, **p < 0.01, ***p < 0.001, ****p < 0.0001 vs. day 2) or Tukey’s test (^#^*p* < 0.05, ^##^*p* < 0.01, ^###^*p* < 0.001, ^####^*p* < 0.0001 for hNEFA vs. nNEFA; ^£^*p* < 0.05, ^££^*p* < 0.01, ^£££^*p* < 0.001, ^££££^*p* < 0.0001 for hNEFA vs. hNEFA + Trolox). Where indicated by square brackets, statistical analysis was performed using one-way ANOVA followed by Tukey’s *post-hoc* test. Only significant differences are shown.

Mitochondrial DNA integrity was likewise restored by Trolox supplementation. Oxidation of the mtDNA D-loop region was significantly reduced in both culture media and follicular walls compared with hNEFA alone (approximately 1.8-fold and 1.7-fold reduction, respectively; *p* < 0.05 and *p* < 0.01; Figure 6E). Importantly, mtDNA oxidation levels in hNEFA + Trolox samples were indistinguishable from those measured under nNEFA conditions in both compartments, as confirmed by the analysis of residual non-oxidized mtDNA (Figure 6E and Supplementary Figure 5). To determine whether the protective effects of Trolox extended to the oocyte mitochondrial compartment, mitochondrial DNA (mtDNA) copy number and mitochondrial membrane potential were evaluated. Oocytes derived from hNEFA + Trolox–treated follicles exhibited a significant increase in mtDNA copy number compared with hNEFA alone (4.3 × 10^5^ vs. 2.0 × 10^5^ copies per oocyte; *p* < 0.05; Figure 7A). However, mtDNA content remained significantly lower than that observed in nNEFA-derived oocytes (*p* < 0.01; Figure 7A), indicating only partial recovery of mitochondrial abundance.

To assess whether reduced mtDNA copy number translated into persistent mitochondrial dysfunction, mitochondrial membrane potential was measured using the JC-1 probe. The red-to-green fluorescence ratio, indicative of mitochondrial membrane potential, was significantly decreased in hNEFA oocytes compared with both nNEFA (*p* < 0.01) and hNEFA + Trolox (*p* < 0.05) groups (Figures 7B,C), reflecting impaired mitochondrial activity under lipotoxic conditions.

In contrast, oocytes from the hNEFA + Trolox group exhibited a red-to-green fluorescence ratio comparable to that observed in nNEFA controls (Figures 7B,C), indicating restoration of mitochondrial membrane potential despite incomplete recovery of mtDNA copy number.

Together, these results indicate that Trolox supplementation normalizes mitochondrial functional activity under hNEFA conditions, even though mitochondrial DNA abundance remains partially reduced.

## 4 Discussion

Female reproductive function is highly sensitive to metabolic status, and altered lipid metabolism is increasingly associated with impaired fertility outcomes (59). Elevated circulating NEFAs are a common feature of conditions characterized by increased adipose tissue lipolysis, including negative energy balance, metabolic stress, and insulin-resistant states (60–65). In these contexts, tissue lipotoxicity appears to depend primarily on the circulating bioavailable NEFA fraction rather than on total lipid intake (66, 110), highlighting the tight connection between metabolic status, nutrient availability, and cellular stress responses within the reproductive system.

In the ovary, excess lipid exposure has been associated with altered follicular development, reduced oocyte quality, and compromised reproductive competence (7, 22, 62, 67–69). However, most available evidence derives from *in vivo* models in which elevated NEFA coexist with systemic confounders such as obesity, endocrine disruption, and chronic inflammation (62, 70–72), conditions often characterized by immune-metabolic dysregulation, or from short-term *in vitro* studies that fail to capture the cumulative metabolic stress occurring during the prolonged course of folliculogenesis (22, 69, 73, 74). Therefore, the direct contribution of sustained NEFA exposure to early follicular development remains incompletely understood. In this context, controlled long-term follicle culture systems represent a powerful experimental approach to isolate follicle-level lipotoxicity and investigate how altered fatty acid availability influences oocyte competence (37, 75, 76), providing a relevant *in vitro* framework to study metabolic and redox responses to nutrient-derived signals.

The present study provides direct evidence that sustained NEFA elevation, mimicking conditions of enhanced adipose tissue lipolysis, exerts lipotoxic effects on follicular function and oocyte competence. Using a long-term three-dimensional *in vitro* folliculogenesis model (37), we show that exposure to moderately elevated NEFA levels dominated by PA, SA, and OA (44, 77–79) induces a progressive oxidative microenvironment that compromises meiotic and developmental competence. Importantly, these effects arise under conditions of prolonged exposure throughout follicular growth rather than an acute lipid overload, supporting the concept that chronic metabolic imbalance may progressively reshape the follicular microenvironment.

In this context, the physiological relevance of the experimental model becomes particularly important. We reproduced a moderate but sustained NEFA elevation (hNEFA 140–210 μM) reflecting conditions associated with increased adipose tissue lipolysis, including negative energy balance and metabolic stress (2, 60, 61, 80, 81).

Also, moderate increases in follicular fluid NEFA concentrations have also been described in women with overweight, obesity, and PCOS, conditions associated with impaired reproductive competence (20, 113).

Fatty acids were supplied in defined proportions and complexed to albumin, respecting physiological fatty acid–albumin (41) and allowing control of the bioavailable NEFA fraction, a key determinant of lipotoxicity (66, 82–84). By controlling fatty acid–albumin ratios in ovine serum (39, 85), the model reproduces physiological NEFA handling while avoiding artifacts of free fatty acid overload. The long-term follicle culture system (18 days) further allows investigation of NEFA effects across early and intermediate folliculogenesis, a developmental window difficult to isolate *in vivo* due to systemic metabolic confounders (86, 87).

A central observation of this study is the dissociation between follicular growth and oocyte competence under sustained hNEFA exposure. Although treated follicles remained viable, exhibited accelerated growth, and reached key developmental milestones such as antrum formation, the functional quality of the enclosed oocytes was markedly impaired. Oocytes derived from hNEFA-exposed follicles displayed reduced meiotic maturation and severely compromised developmental competence following both parthenogenetic activation and IVF. These findings indicate that morphological indicators of follicular development do not necessarily reflect the functional competence of the developing oocyte under altered metabolic conditions. They also suggest differential sensitivity of follicular compartments to metabolic stress; whereby somatic cells maintain structural follicular development while the germ cell remains particularly vulnerable. Similar dissociations between follicular growth and oocyte competence have been reported in large animal models experiencing metabolic stress and negative energy balance (21, 88).

Mechanistically, our results identify oxidative stress as a central mediator linking NEFA exposure to impaired oocyte competence. Sustained NEFA elevation induced progressive ROS accumulation within the follicular microenvironment and altered expression of redox-related genes in somatic cells. Comparable compensatory yet insufficient antioxidant responses have been described in metabolic reproductive disorders such as PCOS (89–92), a condition also characterized by low-grade inflammatory and immune-metabolic alterations, suggesting that increased antioxidant activity may represent an adaptive response to excessive ROS generation rather than restoration of redox balance.

Consistent with this interpretation, increased oxidative burden in our model was associated with molecular signatures of DNA damage. Elevated levels of the oxidative DNA damage marker 8-hydroxy-2′-deoxyguanosine (93) were detected both in the culture medium and within follicular structures, indicating persistent oxidative stress during follicular development. In parallel, hNEFA exposure induced oxidation of the mitochondrial DNA D-loop region, a key regulatory site controlling mitochondrial replication and transcription, reflecting impaired mitochondrial genome integrity (94–96). Notably, these alterations occurred despite preserved follicular morphology and growth dynamics, supporting the notion that oxidative stress selectively compromises oocyte quality. Oocytes, characterized by high metabolic demand and limited antioxidant defenses during early growth stages, appear particularly susceptible to sustained redox imbalance (21, 97, 123).

Consistent with disrupted somatic–germline communication, hNEFA markedly suppressed HAS2 expression in cumulus cells, a marker of cumulus expansion and cumulus–oocyte coupling associated with oocyte competence. Importantly, Trolox supplementation restored HAS2 expression and improved developmental outcomes. These findings align with previous studies demonstrating that disturbances of the follicular microenvironment—including elevated NEFA (98), oxidative stress (99), heat stress (100), and DNA damage (101) can disrupt granulosa and cumulus cell function and thereby compromise oocyte maturation and developmental competence, even when follicular morphology remains preserved.

Mitochondrial function emerged as another critical target of NEFA-induced lipotoxicity. Mitochondrial integrity and mtDNA content are key determinants of oocyte developmental competence (102–104). In the present study, hNEFA exposure significantly reduced oocyte mtDNA copy number. Although Trolox supplementation partially restored mtDNA levels, full normalization was not achieved. Nevertheless, Trolox restored mitochondrial membrane potential, indicating recovery of mitochondrial functional activity despite incomplete restoration of mitochondrial abundance. These observations indicate that mtDNA copy number alone may not a reflect mitochondrial functional status. Mitochondrial quantity and quality are regulated by complex developmental and metabolic cues, and mtDNA content does not necessarily correlate linearly with ATP-generating capacity or immediate developmental competence (102, 104).

Several mechanisms may explain the persistent reduction in mtDNA content. Mitochondrial replication during folliculogenesis occurs within a tightly regulated developmental window in which the mitochondrial pool required for maturation and early embryogenesis is established (48, 105, 106). Perturbations during this phase may therefore produce lasting alterations in mitochondrial endowment, particularly as transcriptional activity progressively declines during oocyte growth. In addition, mtDNA copy number is controlled by selective replication and segregation processes associated with the mitochondrial genetic bottleneck (106, 107).

These observations indicate that oxidative stress represents an important, though not exclusive, mediator of NEFA-induced mitochondrial impairment. While Trolox restored mitochondrial activity, additional NEFA-driven mechanisms—including altered lipid metabolism, ER stress, and changes in organelle dynamics (69, 108) may contribute to the persistent reduction in mtDNA content.

Despite incomplete normalization of mtDNA copy number, Trolox-treated oocytes supported embryo development to the blastocyst stage at rates comparable to those observed under nNEFA conditions. These findings indicate that the residual mitochondrial complement in the hNEFA + Trolox group is sufficient to sustain preimplantation development. This interpretation is consistent with previous evidence indicating that oocytes with reduced mtDNA copy numbers can still progress to the blastocyst stage, whereas a critical mitochondrial threshold is primarily may be particularly relevant for post-implantation development. In a mouse Tfam model, a post-implantation developmental threshold of approximately ~40,000–50,000 mtDNA copies per oocyte has been proposed (109).

, From a translational perspective, the use of Trolox is particularly relevant since vitamin E supplementation has shown beneficial effects on oxidative stress, inflammatory, and metabolic parameters in women with obesity and PCOS, although its direct impact on oocyte competence remains poorly defined (112, 114, 115).

However, species differences and experimental context must be considered, and further *in vivo* validation will be required to determine whether similar thresholds operate under lipotoxic conditions.

## 5 Concluding remarks, limitations, and future perspectives

Overall, this study provides direct evidence that a moderate yet biologically meaningful and sustained increase in bioavailable NEFAs can act as a primary driver of follicular dysfunction rather than merely reflecting systemic metabolic disturbance. Using a physiologically buffered long-term follicle culture system, we show that chronic NEFA exposure across early and intermediate folliculogenesis is sufficient to uncouple follicular growth from oocyte competence. Notably, sustained NEFA exposure over an 18-day period induced persistent alterations in oocyte mitochondrial endowment despite preserved follicular morphology and growth, highlighting the vulnerability of the oocyte to prolonged metabolic stress and the importance of exposure duration in shaping mitochondrial programming.

Mechanistically, oxidative stress emerged as a central mediator of NEFA-induced follicular damage, linking altered lipid availability to nuclear and mitochondrial DNA perturbations within the follicular microenvironment. Antioxidant treatment with Trolox effectively normalized oxidative damage in somatic and germinal compartments and restored mitochondrial functional activity, although mtDNA copy number was only partially recovered. Nevertheless, this residual mitochondrial complement supported embryo development to the blastocyst stage at rates comparable to nNEFA conditions, suggesting that mitochondrial functional competence rather than absolute mtDNA abundance may represent the more immediate determinant of preimplantation developmental capacity under lipotoxic stress. However, consistent with previous evidence (109), embryos may reach the blastocyst stage despite reduced mitochondrial content, whereas higher mitochondrial thresholds may be required for post-implantation development. Further *in vivo* studies will therefore be required to determine whether the persistent reduction in mtDNA copy number observed in the hNEFA + Trolox group has consequences for later developmental stages.

These observations carry important biological and translational implications. They suggest that chronic low-grade lipotoxicity, as may occur in metabolic stress, insulin resistance, or polycystic ovary syndrome, can exert subtle but biologically meaningful effects on oocyte quality that are not predicted by follicular growth or morphology. Such metabolic disturbances are increasingly recognized as part of a broader immune-metabolic imbalance linking nutrition, oxidative stress, and reproductive function. Consequently, strategies aimed at preserving reproductive competence in metabolically compromised environments may need to consider both exposure duration and early preventive interventions targeting key molecular mediators such as oxidative stress.

Some limitations should be acknowledged. Although the ovine long-term *in vitro* folliculogenesis model provides a controlled and translationally relevant platform, it cannot fully recapitulate the systemic complexity of human metabolic disorders or the dynamic maternal–embryo interactions occurring *in vivo*. Nevertheless, this system allowed isolation of direct follicle-level NEFA effects independently of confounding factors such as obesity, endocrine disruption, and inflammation. Future studies should extend these findings to complementary *in vitro* and *in vivo* systems and explore whether nutritionally derived bioactive compounds with antioxidant and metabolic regulatory properties can more effectively preserve oocyte mitochondrial integrity and long-term developmental competence in metabolically compromised environments.

## Data Availability

The original contributions presented in the study are included in the article/Supplementary material, further inquiries can be directed to the corresponding authors.
