# Supplementary material for: Moderate NEFA reprogram early follicular development and oocyte competence: evidence for a targetable redox mechanism
Source: Front Nutr. 2026 Jun 17;13:1840637. doi: 10.3389/fnut.2026.1840637 (PMC13318601; doi:10.3389/fnut.2026.1840637)
Supplement: Supplementary file 1 [file Image_1.pdf]

Supplementary Material

Supplementary Figure 1

A

	Degenerated follicles (%±SD)	
**BSA**	0 mg/mL	1.6 mg/mL
	10.6 ± 2.0	13.0 ± 4.4

B

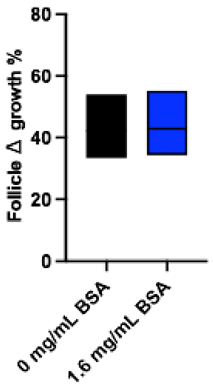

C

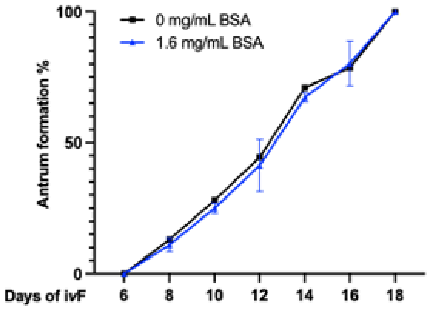

Supplementary Figure 1. Toxicity assessment of 1.6 mg/mL BSA in the NEFA-free follicle cultural system. PAfs were cultured in NEFA-free medium either in the absence (CTRL, 0 mg/mL) or presence of 1.6 mg/mL BSA. Degeneration rate (A), follicular growth rate (Δ) (B) and antrum formation over time (C) were recorded after 18 days of 3D in vitro culture. Data (mean ± SD) represent a total of 60 PAfs pooled from three independent biological replicates.
