# Supplementary material for: Moderate NEFA reprogram early follicular development and oocyte competence: evidence for a targetable redox mechanism
Source: Front Nutr. 2026 Jun 17;13:1840637. doi: 10.3389/fnut.2026.1840637 (PMC13318601; doi:10.3389/fnut.2026.1840637)
Supplement: Supplementary file 2 [file Image_2.pdf]

Supplementary Material

Supplementary Figure 2

**nNEFA**			
**RSV dose (µM)**	**Degenerated Follicles (%; SD)**	**Trolox dose (µM)**	**Degenerated Follicles (%; SD)**
0	13.0 ± 7.0	0	13.0 ± 7.0
12.5	13.0 ± 1.0	37.5	12.7 ± 5.5
25	17.0 ± 3.0	75	13.0 ± 4.6
50	43.0 ± 7.0^{abc}	150	14.7 ± 11.6
100	47.0 ± 3.0^{abcd}	300	81.3 ± 4.2^{abcd}

Supplementary Figure 2. Dose–response curves of RSV and Trolox in long-term PAfs 3D cultures. RSV was tested at 0, 12.5, 25, 50, and 100 µM, and Trolox at 0, 37.5, 75, 150, and 300 µM. After 18-days of incubation, early antral follicles (EAfs) were classified as healthy or degenerated. Data (mean ± SD) represent a total of 90 follicles per dose, pooled from three independent biological replicates. Statistical analysis was performed using two-way ANOVA. Values with different superscript letters differ significantly ($p < 0.05$). For each antioxidant, superscripts denote comparisons among concentrations within the same treatment. Specifically, for RSV: (a) vs 0 µM; (b) vs 12.5 µM; (c) vs 25 µM; (d) vs 50 µM. For Trolox: (a) vs 0 µM; (b) vs 37.5 µM; (c) vs 75 µM; (d) vs 150 µM. Only significant differences are indicated.
