# Supplementary material for: Moderate NEFA reprogram early follicular development and oocyte competence: evidence for a targetable redox mechanism
Source: Front Nutr. 2026 Jun 17;13:1840637. doi: 10.3389/fnut.2026.1840637 (PMC13318601; doi:10.3389/fnut.2026.1840637)
Supplement: Supplementary file 3 [file Image_3.pdf]

Supplementary Material

Supplementary Figure 3

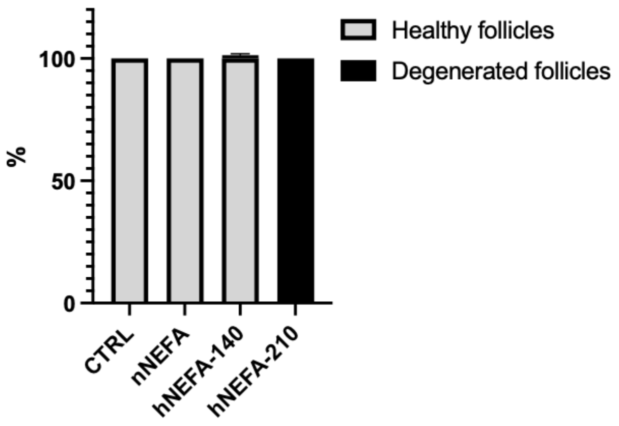

Supplementary Figure 3. Early time-point assessment of follicle viability under increasing NEFA loads. Morphological evaluation of follicular degeneration at 8 days of *iv*F culture. Data (mean ± SD) represent a total of 90 follicles pooled from three independent biological replicates. Only significant differences are indicated.
