# Supplementary material for: Moderate NEFA reprogram early follicular development and oocyte competence: evidence for a targetable redox mechanism
Source: Front Nutr. 2026 Jun 17;13:1840637. doi: 10.3389/fnut.2026.1840637 (PMC13318601; doi:10.3389/fnut.2026.1840637)
Supplement: Supplementary file 4 [file Image_4.pdf]

Supplementary Material

Supplementary Figure 4

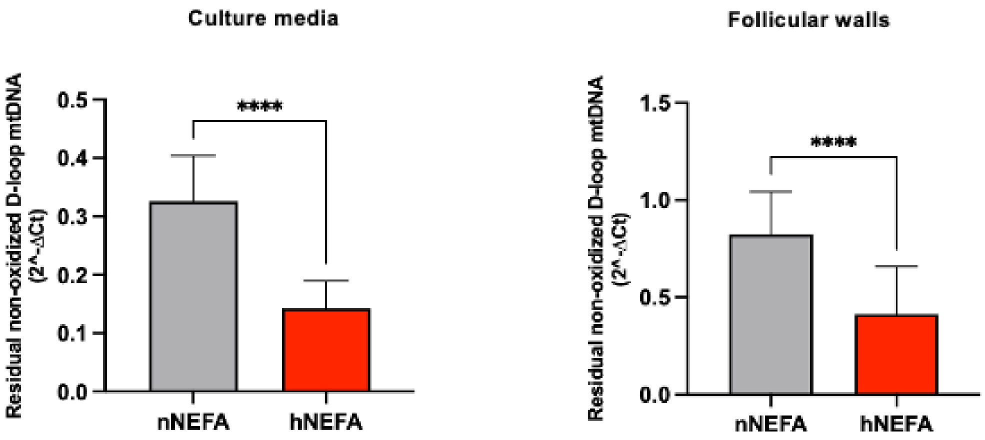

Supplementary Figure 4: Oxidation of the mtDNA D-loop region, expressed as residual non oxidized mtDNA. Data (mean ± SD) represent a total of 90 follicles pooled from three independent biological replicates. t—test was applied to calculate statistically significant differences with **** $p<0.0001$.
