# Supplementary material for: Moderate NEFA reprogram early follicular development and oocyte competence: evidence for a targetable redox mechanism
Source: Front Nutr. 2026 Jun 17;13:1840637. doi: 10.3389/fnut.2026.1840637 (PMC13318601; doi:10.3389/fnut.2026.1840637)
Supplement: Supplementary file 5 [file Image_5.pdf]

Supplementary Material

Supplementary Figure 5

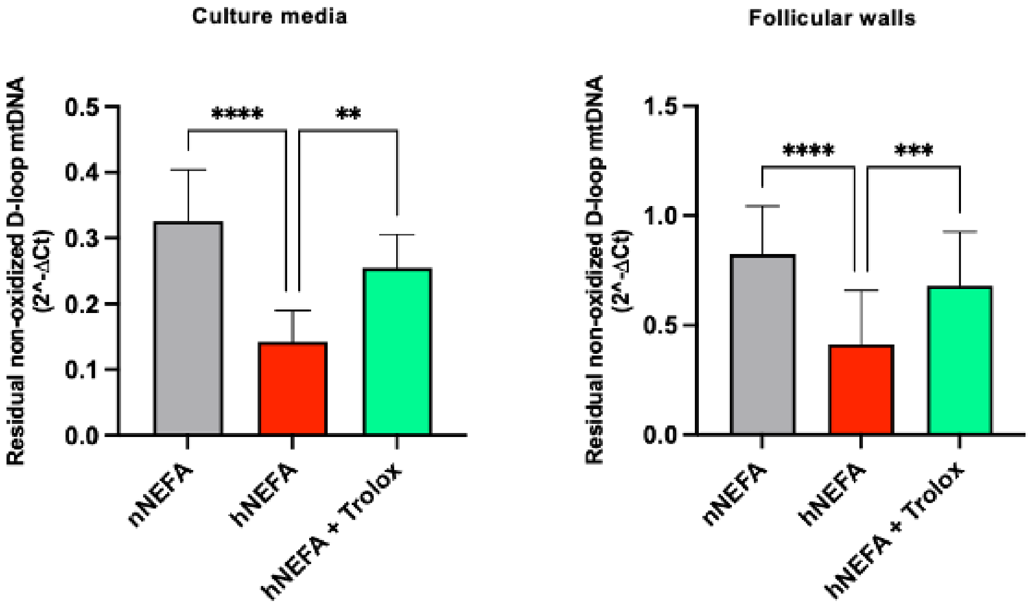

Supplementary Figure 5: Trolox restores residual non-oxidized mtDNA D-loop fraction in follicular walls and culture media under hNEFA conditions. Oxidation of the mtDNA D-loop region, expressed as residual non oxidazed mtDNA. Data (mean ± SD) represents a total of 90 follicles pooled from three independent biological replicates. Statistical analysis was performed using one-way ANOVA followed by Tukey's post hoc test ($^{**}p < 0.01$, $^{***}p<0.001$, $^{****}p<0.0001$).
